# Cdk4 and Nek2 Signal Binucleation and Centrosome Amplification in a Her2+ Breast Cancer Model

**DOI:** 10.1371/journal.pone.0065971

**Published:** 2013-06-11

**Authors:** Mary Kathryn Harrison Pitner, Harold I. Saavedra

**Affiliations:** Department of Radiation Oncology, Emory University School of Medicine, Atlanta, Georgia, United States of America; Florida International University, United States of America

## Abstract

Centrosome amplification (CA) is a contributor to carcinogenesis, generating aneuploidy, and chromosome instability. Previous work shows that breast adenocarcinomas have a higher frequency of centrosome defects compared to normal breast tissues. Abnormal centrosome phenotypes are found in pre-malignant lesions, suggesting an early role in breast carcinogenesis. However, the role of CA in breast cancers remains elusive. Identification of pathways and regulatory molecules involved in the generation of CA is essential to understanding its role in breast tumorigenesis. We established a breast cancer model of CA using Her2-positive cells. Our goal was to identify centrosome cycle molecules that are deregulated by aberrant Her2 signaling and the mechanisms driving CA. Our results show some Her2+ breast cancer cell lines harbor both CA and binucleation. Abolishing the expression of Cdk4 abrogated both CA and binucleation in these cells. We also found the source of binucleation in these cells to be defective cytokinesis that is normalized by downregulation of Cdk4. Protein levels of Nek2 diminish upon Cdk4 knockdown and vice versa, suggesting a molecular connection between Cdk4 and Nek2. Knockdown of Nek2 reduces CA and binucleation in this model while its overexpression further enhances centrosome amplification. We conclude that CA is modulated through Cdk4 and Nek2 signaling and that binucleation is a likely source of CA in Her2+ breast cancer cells.

## Introduction

Theodor Boveri’s work published in 1914 was the first to hypothesize a correlation between abnormal centrosome numbers, aneuploidy, and tumorigenesis [1]. Almost 100 years later, the questions surrounding this correlation are still being pursued. Centrosomes play a crucial role in maintaining euploidy; the two mitotic centrosomes direct the formation of a bipolar spindle and allow equal segregation of chromosomes into daughter cells [2]. Centrosome amplification (CA), the acquisition of three or more centrosomes within a cell, is often observed in human cancers and has been shown to contribute to multipolar mitoses, aneuploidy, and chromosomal instability [3]–[6]. There is a growing body of evidence showing that a majority of solid tumors and some hematopoietic cancers harbor cells with centrosome abnormalities, either numerical or structural [7]. Observations in breast tumors show that adenocarcinoma cells have a much higher occurrence of centrosome defects, including amplification of number, increased volume, and supernumerary centrioles, when compared to normal breast tissue [8], [9]. Similar phenotypes can also be found in premalignant lesions and pre-invasive *in situ* ductal carcinoma, suggesting that these aberrations influence early breast carcinogenesis [9]–[11]. Although the role played by CA in mammalian tumorigenesis remains a mystery, major discoveries have been made. Among these is the discovery that ectopic expression of centrosome and mitotic regulatory kinases results in CA and tumorigenesis in *Drosophila* [12], [13]. Another finding is that low-level aneuploidy caused by interference with the spindle assembly checkpoint initiates mouse tumors [14], [15], and that CA is capable of generating low levels of aneuploidy [16]. CA is also known to generate more severe forms of aneuploidy, including tetraploidy, through generating multipolar spindles [17]. Although tetraploidy is selected against in checkpoint-proficient cells [16]–[18], it contributes to carcinogenesis in p53-deficient mammary epithelial cells [19]. It has been reported that the absence of p53 allows transient tetraploidy in a small subset of cell lines [18].

The centrosome duplication cycle is coordinated with the cell cycle, such that it occurs only once per mitosis [20]. The biology of cell cycle regulation has been well studied [21], [22], and it is known that the faithful regulation of its phases, G_1_, S, and G_2_/M, is important to cancer prevention [23]. More recent work has shown that there are many cell cycle regulatory proteins (including the cyclins, Cdks, CKIs, and E2Fs) that associate with the centrosome cycle and seem to play a role in centrosome homeostasis [17], [24], [25]. A large number of these proteins have also been reported as deregulated in cancer. For example, Cdk2 and Cdk4 are two proteins central to the coordination of the cell and centrosome duplication cycles. It has been previously shown that Cdk4 is a regulator of centrosome duplication [26], [27], that the cyclin D1/Cdk4 complex contributes to p53-null- and Ras-driven CA [26], [28] and is important in Her2 mitogenic signaling [29]–[31]. Many studies implicate Cdk2 as a key regulator in several centrosomal functions including: centrosome duplication [17], [32]–[35], CA in p53-negative breast cancer cells [36] and p53-null mouse embryonic fibroblasts [26], [37], [38], and cells expressing the E7 viral oncoprotein [39]. Because ablation of Cdk2 or Cdk4 suppresses Her2-driven mammary tumors [31], [40], [41] and signals CA, the two Cdks may represent important links between CA and tumorigenesis.

Her2/Neu, also known as ErbB2, a receptor tyrosine kinase, induces a complex signaling network upon binding its co-receptors, among these activated signals is the well-studied Ras-activated mitogen activated protein kinase (MAPK) pathway [30]. While rarely mutated in human cancers, wild-type Her2 is often found amplified at the gene level or overexpressed at the protein level. The oncoprotein is overexpressed in approximately 30% of breast tumors, and hyper-activates and deregulates its downstream signaling networks, including the G_1_/S cell cycle phase via high levels of cyclin D and active cyclin D/Cdk4/6 complexes [29]. Cyclin D1 and its catalytic partners Cdk4/Cdk6 have been shown to be required for Her2-induced transformation [41]–[44], but the mechanism driving this phenotype remains unknown. There are studies suggesting association between Her2 over-expression and CA in breast tumors [9], [45], and one showing that mammary tumors in MMTV-*Neu* mice display CA [46], but the molecular contribution of Cdk2 and Cdk4 to Her2/Neu-mediated CA has yet to be elucidated.

It has long been thought that CA is a mechanism that leads to chromosomal instability [17], [47], a distinguishing feature of cancer cells, through abnormal mitoses. A recent study provided a direct link between CA and chromosomal instability, showing that extra centrosomes are sufficient to promote chromosome gains and losses during a pseudobipolar mitosis through a multipolar spindle intermediate [16]. Increased centrosome defects are directly proportional to chromosome aberrations in breast tumors, suggesting that CA is a driver of aneuploidy [5], [48]. Because aneuploidy is transforming, and correlates with chemoresistance in tumors [49], finding agents that can prevent or suppress CA and the active generation of chromosomal instability in tumors is essential to cancer control. Direct evidence showing that CA transforms primary mammary epithelial cells is lacking, and necessitates the identification of oncogene-driven centrosomal regulatory molecules signaling CA. This study elucidates mechanisms responsible for CA in a Her2+ breast cancer model. Due to extensive evidence that Cdk2 and Cdk4 are important genetic links between CA, mitotic errors, and transformation, we explored their role as major regulators of CA in Her2+ breast cancer cells. Our results illustrate that the presence of CA, binucleation and defective cytokinesis requires Cdk4 but not Cdk2. In addition, we found that Nek2 may be a downstream target of Cdk4 that regulates its expression and mediates its role in binucleation and CA.

## Materials and Methods

### Cell Culture

SKBr3 (ATCC, Manassas, VA, USA, HTB-30) and HCC1954 (ATCC, CRL-2338) cells were maintained under proliferating conditions in RPMI media (Sigma, St. Louis, MO, USA, R8758) supplemented with 10% fetal bovine serum (FBS) and 1% Penicillin/Streptomycin antibiotics (Gibco, Carlsbad, CA, USA, 15140). MCF10A (ATCC, CRL-10317) cells were maintained in DMEM/F-12 media (Gibco, 12500-096) supplemented with 10% FBS, 1% Penicillin/Streptomycin, NaHCO_3_, HEPES, 10 µg/ml Insulin, 20 ng/ml EGF, 0.5 µg/ml hydrocortisone, and 100 ng/ml cholera toxin. For serum arrest/release experiments, cells were cultured in 0.2% FBS for 72 hours under serum arrest conditions, and then released through the addition of serum. All cell lines screened, but not used for further investigation in this manuscript originated from the ATCC.

### Lentiviral Infections

Lentiviral infections were done to create stable cell lines. The Expression Arrest lentiviral shRNA pLKO.1 vector system was used from Open Biosystems (Thermo Scientific, Waltham, MA, USA). 293T cells were co-transfected with 1.8 µg target shRNA construct, 1.8 µg pHRCMV8.2ΔR, and 0.18 µg pCMV-VSVG helper plasmids. Viral supernatant from 293T cell culture media was collected three times in 8-hour increments beginning 48 hours after transfection. Target cell lines were infected with viral supernatant and 10 mg/ml polybrene. Forty-eight hours after the final infection, selection was begun in complete media containing 2 ug/ml puromycin (Sigma, p9620). Resistant cells were assayed for knockdown of the target gene by Western blot.

### Transfections

Transient transfection of siRNAs was done using Lipofectamine 2000 (Invitrogen, Carlsbad, CA, USA, 11668). siRNAs against Cdk2, Cdk4, and Nek2 were designed and purchased from IDT (IDT, Coralville, IA, USA). As a negative control, Silencer Negative Control #1 RNA (Ambion, Carlsbad, CA, USA, 4611) was transfected. Transfections were performed as per the manufacturer’s protocol; 72 hours after transfection the cells were used to prepare cell lysates for western blots or fixed in preparation of immunofluorescent staining. siRNA sequences are included as supplementary information (Table S1). Nek2 was subcloned into the pMONO-Hygro-GFP plasmid (Invivogen, San Diego, CA, USA, pmonoh-gfp) by the Emory DNA Custom Cloning Core Facility. Transfection of the pMONO-Hygro-GFP-Nek2 plasmid was done using TransIT-2020 Transfection Reagent (Mirus, Madison, WI, USA, MIR5404) according to the manufacturer’s instructions. HCC1954 shpLKO.1; GFP-Nek2 and HCC1954 shCdk4-4; GFP-Nek2 cells were maintained in RPMI media (Sigma, R8758) supplemented with 10% fetal bovine serum (FBS), 1% Penicillin/Streptomycin antibiotics (Gibco, 15140), 2 ug/ml puromycin and 25 ug/ul hygromycin (Sigma, h0654).

### Immunofluorescence

Immunofluorescence was performed following our published protocols [26], [28]. Proliferating cells plated in four chamber slides (Thermo Scientific, Waltham, MA, USA 154526) were fixed in cold 4% paraformaldehyde, washed in PBS, permeabilized in a 0.1% NP40-PBS solution, and blocked in 10% normal goat serum (Invitrogen, 50-062Z). Centrosomes and cytoskeletal structures were stained overnight at 4°C with antibodies against pericentrin (abcam, Cambridge, UK, ab4448) or α-tubulin (Santa Cruz, Santa Cruz, CA, USA, sc-32292), respectively. Alexa Fluor 488 goat anti-rabbit (Invitrogen, A11008) and Alexa Fluor 488 goat anti-mouse (Invitrogen, A11001) conjugated secondary antibodies were used, respectively. Cells were counterstained with 4′,6-diamidino-2-phenylindole (DAPI).

### Western Blotting

Western blotting was performed according to our published protocols [26], [50]. Antibodies used in western blotting experiments are as follows: Her2 (Cell Signaling, Beverly, MA, USA, 2165), Cyclin D1 (Cell Signaling, 2922), Nek2 (BD Biosciences, San Jose, CA, USA, 610593), phospho-NPM (Thr199) (Cell Signaling, 3541), NPM (Invitrogen, 32-5200), GFP (abcam, ab290), β-actin (Cell Signaling, 4970), Cdk2 (Santa Cruz, sc-163), and Cdk4 (Cell Signaling, 2906).

### Image Acquisition

Slides were analyzed using a Zeiss Axioplan II (Zeiss, Oberkochen, Germany) microscope with a Plan-Apochromat 63× oil immersion objective. Images were taken using the Axiocam HRC and Zeiss Axiovision software. Confocal images were acquired with a Zeiss LSM 510 META point scanning laser confocal microscope mounted on a Zeiss Axioplan II upright microscope equipped with a Plan-Apochromat 20× objective. Images were captured on the Zeiss Image Browser. All fixed samples were mounted in Fluoromount-G mounting medium (Southern Biotech, Birmingham, AL, USA) and were analyzed at room temperature.

### Live Microscopy

Proliferating cells were plated on an eight-chambered #1.5 German coverglass system (LabTek II, 155409). Live cells were imaged at 20x on the PerkinElmer Ultra View Spinning Disk (PerkinElmer, Waltham, MA, USA) microscope at 37°C and 5% CO_2_, with a differential interference contrast (DIC) filter. Images were captured every five minutes for at least twenty-four hours, and compiled into movies for analysis. All image capture and analysis was done using the Volocity 3D Image Analysis Software (PerkinElmer).

### BrdU Analysis

BrdU incorporation analysis was performed according to our published protocols [26]. Pulsed cells were fixed and incubated overnight at 4°C with anti-BrdU antibody (Calbiochem, Billerica, MA, USA, NA61), then for 1 hour at room temperature with Alexa Fluor 555 anti-mouse secondary antibody (Invitrogen, A21422) and counterstained with DAPI.

### Flow Cytometry

Cells were dissociated from culture plates using Accutase (Sigma, A6964) and collected by spinning down in 15 ml conical tubes. Cells were washed in cold 1X PBS and fixed in cold 70% Ethanol. After fixation, cells were treated with with 500 µl RNase (Sigma, R5125) and stained with 500 µl propidium iodide (Sigma, P4170) for 45 minutes. Cells were transferred to meshed cap Falcon tubes for FACS analysis. FACS analysis was performed on a Benton-Dickinson LSRII.

## Results

### Establishing a Model for the Study of Centrosome Amplification in Breast Cancer

**Figure 1:**
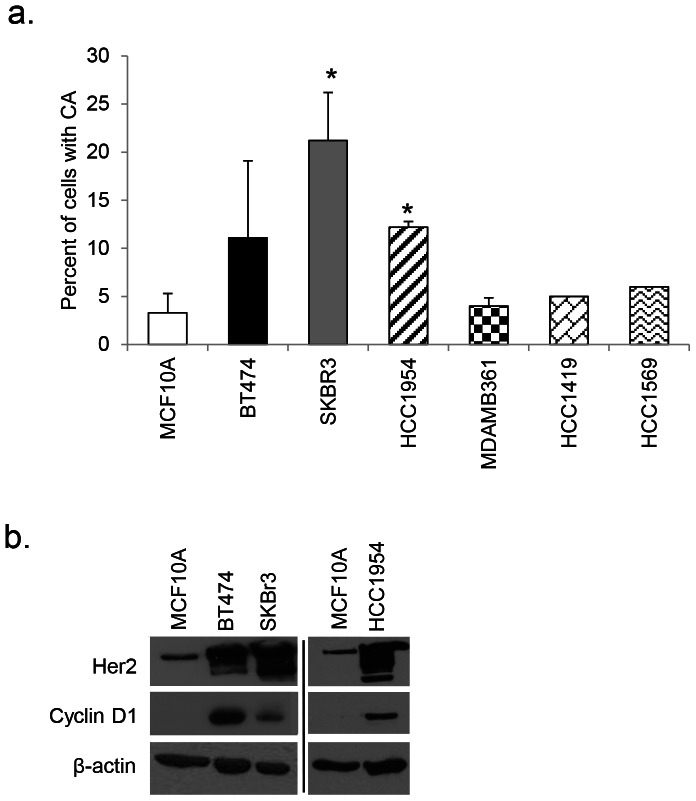
Her2+ cells display CA. (a) Centrosome amplification (CA) was measured by staining proliferating cells plated in four-chambered microscopy slides with an antibody against pericentrin and counterstaining with DAPI. Independent experiments were done three times using 200 cells per experiment. Graphs show the percent of cells with CA. Statistical significance was addressed using a T-test (* = p≤0.05). (b) Protein lysate was collected under starvation conditions. MCF10A and Her2+ breast cancer cell lines were probed with antibodies against Her2, and cyclin D1; β-actin was used as a loading control. Western blot results show two separate gels; different exposures are commensurate with protein abundance.

In order to establish a breast cancer cell model of CA, we screened several established breast cell lines of varying molecular subtypes for the presence of CA. We observed that SKBr3 and HCC1954 Her2+ER-PR- breast cancer cell lines harbor significantly higher percentages of CA in comparison to MCF10A control cells (Figure 1a). BT474 showed elevation in CA approaching statistically significance (p<0.07); because these cells grow in multiple layers precise calculation of CA was difficult. Analysis from CCLE and COSMIC databases, as well as results from the literature show that there are no mutations detected in HRAS, KRAS, or NRAS in MCF10A or cells displaying CA. Whereas previous reports demonstrate correlation between Her2 overexpression and CA using biopsied patient tissue, our study focuses on a Her2+ cell line experimental model. Following our initial screen, we determined that SKBr3 and/or HCC1954 would be used for further modeling of CA in breast cancer.

### Centrosome Amplification in Her2+ Cells is Abrogated with Silencing of Cdk4

It has been shown that amplification of the Her2 gene is significantly correlated with centrosome abnormalities in breast tumors [9], [45], [46], which could be indicative of a role for CA in the formation and/or progression of Her2+ breast cancer. Based on previous work [51], we sought to understand the role of the G_1_ Cdks in a Her2-mediated CA model. First, we found overexpression of cyclin D1 in BT474, SKBr3, and HCC1954 compared to MCF10A control cells (Figure 1b).

**Figure 2:**
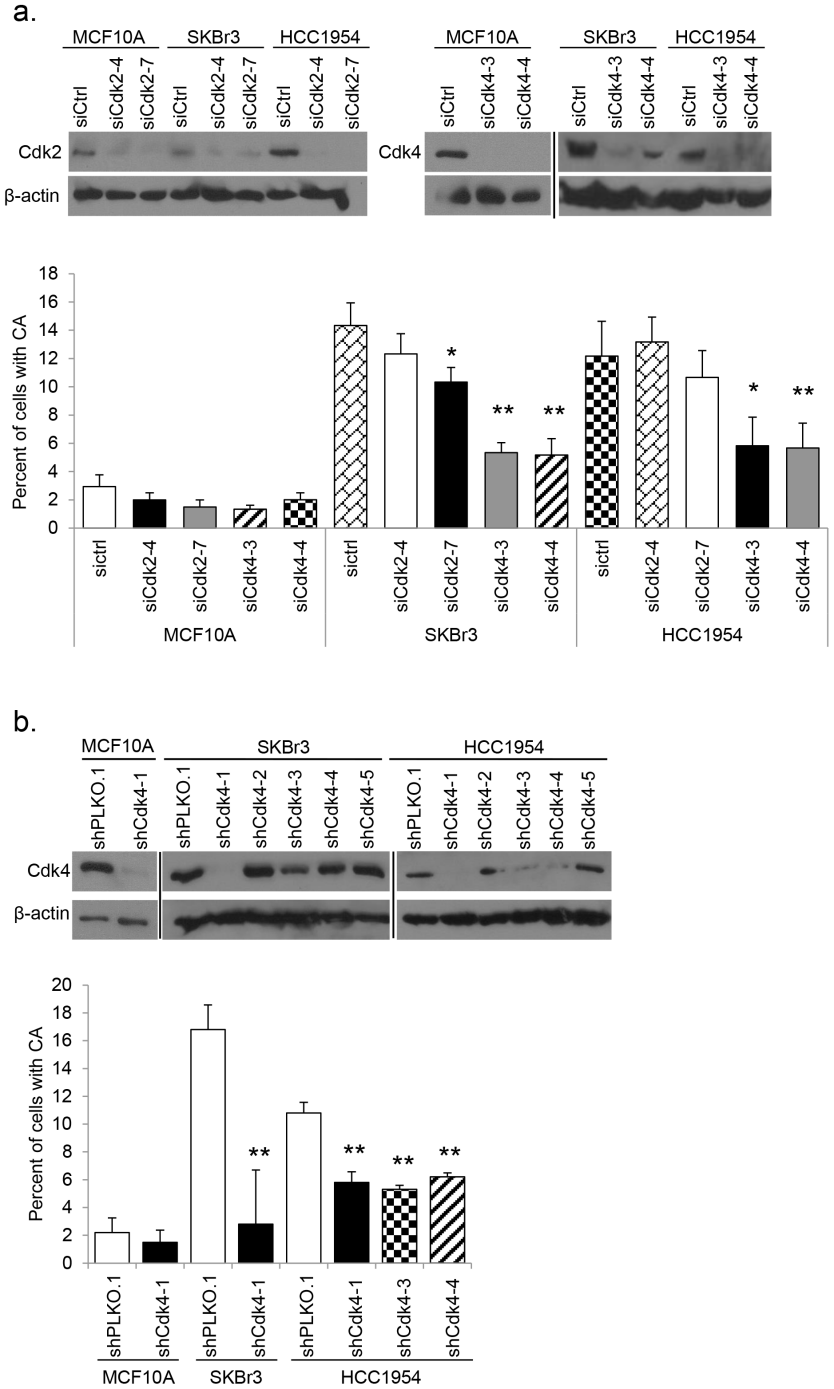
CA in Her2+ cells is mediated by Cdk4. (a) siRNAs against Cdk2 and Cdk4 were transfected into target cell lines; scrambled siRNA was used as a control. siRNA knockdown was confirmed by western blot using antibodies against Cdk2 and Cdk4; β-actin was used as a loading control. Western blot results show three separate gels; different exposures are commensurate with protein abundance. The number of centrosomes in proliferating cells was measured as described in Figure 1a. Statistical significance was addressed using a T-test (* = p≤0.05; ** = p≤0.01). (b) Lentiviral shPLKO.1 control and shCdk4 vectors were used to infect MCF10A, SKBr3, and HCC1954 and create stable cell lines via puromycin selection. Independent lentiviral clones were screened in each cell line; knockdown was confirmed by western blot using an antibody against Cdk4; β-actin was used as a loading control. Western blot results show three separate gels; different exposures are commensurate with protein abundance. Centrosome amplification was measured in cell lines where knockdown was successful as described in Figure 1a. Statistical significance was addressed using a T-test (** = p≤0.01).

Next, we targeted both Cdk2 and Cdk4 in non-tumorigenic and Her2+ breast cancer cells using independent siRNA duplexes. We confirmed knockdown of each gene by Western blot (Figure 2a). CA analysis was done on proliferating cell populations with validated siRNA knockdown. In MCF10A cells, no difference was seen in the percentage of CA between scrambled control and siCdk2 or siCdk4 transfected cells. Both SKBr3 and HCC1954 cell lines showed little to no significant difference in the percentage of CA upon knockdown of Cdk2. However, knockdown of Cdk4 induced a dramatic decrease in CA in both Her2+ cell lines (Figure 2a). As siRNA knockdown is transient, we endeavored to establish stable cell lines expressing shCdk4 (Figure 2b). Mirroring the observations seen using siRNA, stable knockdown of Cdk4 resulted in a significant reduction in the percentage of CA in Her2+ cell lines (Figure 2b). In conclusion, we showed that inhibition of Cdk2 has a nominal effect on the CA phenotype in a Her2+ model of CA and that Cdk4 is a more influential mediator of the phenotype.

To ensure that knocking down Cdk4 did not induce cell cycle arrest, and as a byproduct, a reduction in CA due to lack of cell proliferation, we performed several cell cycle analysis experiments. To make certain shCdk4 cells were progressing through the cell cycle, HCC1954 shPLKO.1 and shCdk4-4 cells were serum arrested for 72 hours. Upon the addition of serum, starting at time zero hours, we harvested cells for cell cycle analysis every 6 hours for 24 hours. Flow cytometry results indicate that shCdk4 cells follow a very similar cell cycle pattern to control cells. A modest difference was seen in the S phase fraction at 18 hours post serum addition, but by 24 hours there is no significant difference (Table 1). This data suggests that loss of Cdk4 affects neither cell cycle entry after serum starvation nor proliferation.

**Table 1:** Knockdown of Cdk4 does not affect cell cycle profiles.

Cell Line	Hours	Percent of G_1_ cells (SD)*	Percent of S cells (SD)*	Percent of G_2_ cells (SD)*
	0	73.4 (3.1)	9.9 (1.2)	16.4 (4.1)
	6	68.9 (3.0)	12.2 (1.2)	18.2 (3.3)
HCC1954 shPLKO.1	12	71.3 (5.4)	4.7 (1.9)	23.9 (7.5)
	18	59.2 (2.8)	24.4 (2.5)	16 (2.9)
	24	35.3 (0.6)	35.3 (9.6)	28.9 (10.1)
	0	76 (0.1)	6.8 (3.8)	17 (4.1)
	6	72.7 (2.8)	10.1 (0.5)	16.8 (2.4)
HCC1954 shCdk4-4	12	74.8 (2.5)	4.7 (0.5)	20.2 (3.1)
	18	68 (1.7)	13.7 (1.4)	17.9 (2.6)
	24	25.7 (7.5)	29.5 (5.3)	44.3 (1.5)

Results are pooled from two independent experiments.

* p-value is calculated by T-test.

To further investigate the S phase fraction of these cells, BrdU incorporation assays were used as described in the Materials and Methods section of this manuscript. We confirmed that stable shCdk4 cell lines were not deficient in S phase cells; our results showed control and shCdk4 cells had similar percentages of cells that stained positive for BrdU (Table 2). These results demonstrate that silencing Cdk4 does not affect the cell cycle, but rather, selectively affects the CA phenotype in this Her2+ breast cancer model.

**Table 2:** Knockdown of Cdk4 does not affect the fraction of cells in S phase.

Cell Line	Percent of BrdU+ cells	SD	p-value*
HCC1954 shPLKO.1	34.1	12.03	
HCC1954 shCdk4-1	34.9	4.35	0.91
HCC1954 shCdk4-3	31.7	12.34	0.83
HCC1954 shCdk4-4	29.8	7.14	0.63

Results are from three independent experiments.

* p-value is calculated by T-test.

### Her2+ Cells Show a High Percentage of Binucleation, Which is Reduced upon Silencing of Cdk4

**Figure 3:**
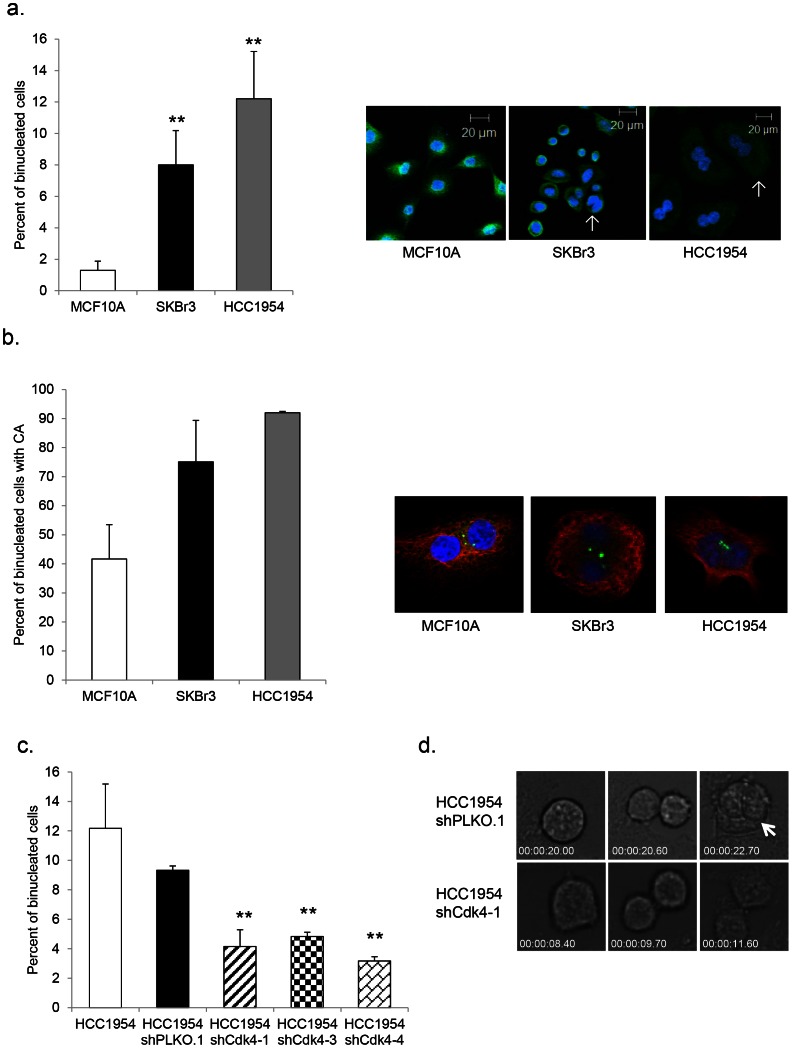
Her2+ breast cancer cells display elevated percentages of binucleation and cytokinesis defects. (a) Binucleation was measured in MCF10A, SKBr3, and HCC1954 parental cell lines by fixing, processing, and staining proliferating cells with an antibody against α-tubulin and counterstaining with DAPI. Arrows indicate binucleated cells. Independent experiments were done three times using 200 cells per experiment. Graphs show the percent of binucleated cells. Statistical significance was addressed using a T-test (* = p≤0.05; ** = p≤0.01). (b) The percentage of CA in binucleated cells was measured by fixing, processing, and staining proliferating cells with antibodies against pericentrin and α-tubulin and counterstaining with DAPI. Independent experiments were done two times using 200 cells per experiment. The percentage of binucleation was measured as described in (a). (c) Binucleation was measured in HCC1954 parental, HCC1954 shPLKO.1 control, and HCC1954 shCdk4 cells as described in (a). (d) Still panels were captured from live cell imaging video of HCC1954 shPLKO.1 and shCdk4-1 to analyze the formation of binucleates in a proliferating population. Arrow indicates a binucleate resulting from failed cytokinesis.

There are several different mechanisms that may generate CA, including, but not limited to de novo centriole assembly, centriole reduplication, and cytokinesis failure [52]. Interestingly, we observed a phenotype of binucleation in HCC1954 and SKBr3 cells compared to MCF10A control cells using antibodies against α-tubulin and DAPI, to image the cytoskeleton and nucleus, respectively (Figure 3a). This phenotype correlates with CA; cells that were binucleated were also overwhelmingly positive for CA. As shown in Figure 3a and 3b, proliferating SKBr3 cells displayed 8.0% binucleation and 75.1% of these cells also harbored CA; 12.2% of proliferating HCC1954 cells were binucleated, and 91.9% of the binucleated population had CA. There is a reasonable amount of data in the literature suggesting a mechanistic link between binucleation and centrosome abnormalities [18]. The source of a potential cytokinetic defect causing binucleation and CA could span the entirety of the cell cycle. Deregulation could lie at the level of molecules directly involved in cytokinesis or could lie upstream in molecules that regulate the cell cycle and its progression.

To ascertain the role of Cdk4 in generating binucleated Her2+ cells, we compared control and shCdk4 HCC1954 cells via microscopy. Results showed a significant decrease in binucleation in all three independent populations compared to vector control and parental cells (Figure 3c), suggesting a correlation between CA and binucleation.

For higher resolution and to reveal additional cellular mechanisms, we employed live cell imaging techniques. Using proliferating HCC1954 shCdk4-1 cells and their respective control, images were captured every 5 minutes over the course of 24 hours, and then pooled to create movies of a field of cells dividing over time. The results were rather striking; we observed cells attempting to undergo mitosis, failing, and resulting in binucleation (Figure 3d). While these events were rare, we noted them in both control and shCdk4-1 cells; however, there is a clear and significant difference in the percentage of these events between the two cell populations. Control cells present with 2.4% of attempted mitoses ending in observed novel binucleation, while in shCdk4-1 cells, only 0.4% of attempted mitoses ended in this way (Table 3). This data provides cytokinesis failure as a discernible mechanism for Cdk4 mediation of binucleation and CA in a Her2+ model.

**Table 3:** Knockdown of Cdk4 reduces percentage of failed mitosis ending in binucleation.

Cell Line Group	N	Observed Phenotype (%)		p-value*
		Binucleated	Non-binucleated	
**HCC1954 shPLKO.1**	645	15 (2.3)	630 (97.7)	0.0089
**HCC1954 shCdk4-1**	485	2 (0.4)	483 (99.6)	

Results are pooled from three independent experiments.

* p-value is calculated by Chi-square test.

### Loss of Nek2 Mimics the Loss of Cdk4 and Correlates with Reduced Centrosome Amplification and Binucleation in Her2+ Breast Cancer Cells

A previous study published by our laboratory screened a broad panel of cell and centrosome cycle regulators in MCF10A cells stably expressing H-Ras^G12V^ or H-Ras^G12V^ and c-Myc [28]. While this study identified genes that influenced a CA phenotype, no mechanism was revealed. The screen pulled down several interesting targets, one of which, Nek2, seemed particularly significant in light of the observed binucleation phenotype in Her2+ cells. Nek2, a NIMA-related cell cycle dependent protein kinase, is normally involved in centrosome separation at the onset of mitosis through phosphorylation of centrosomal proteins [53], [54], its activity peaking in S and G_2_ phases [55]. Nek2 levels have been found to be elevated in human breast cancer [56]. Other proteins reported to be involved in the formation of CA were also found to be deregulated in Her2+ cell lines. Nucleophosmin (NPM) is a negative suppressor of centrosome licensing; it is a target of Cdk2 and Cdk4 phosphorylation during duplication initiation and a known suppressor of CA [25], [57]. Deregulated NPM has been shown to mediate CA in other systems, including p53^−/−^ mouse embryonic fibroblasts through Cdk2 and Cdk4 [26].

**Figure 4:**
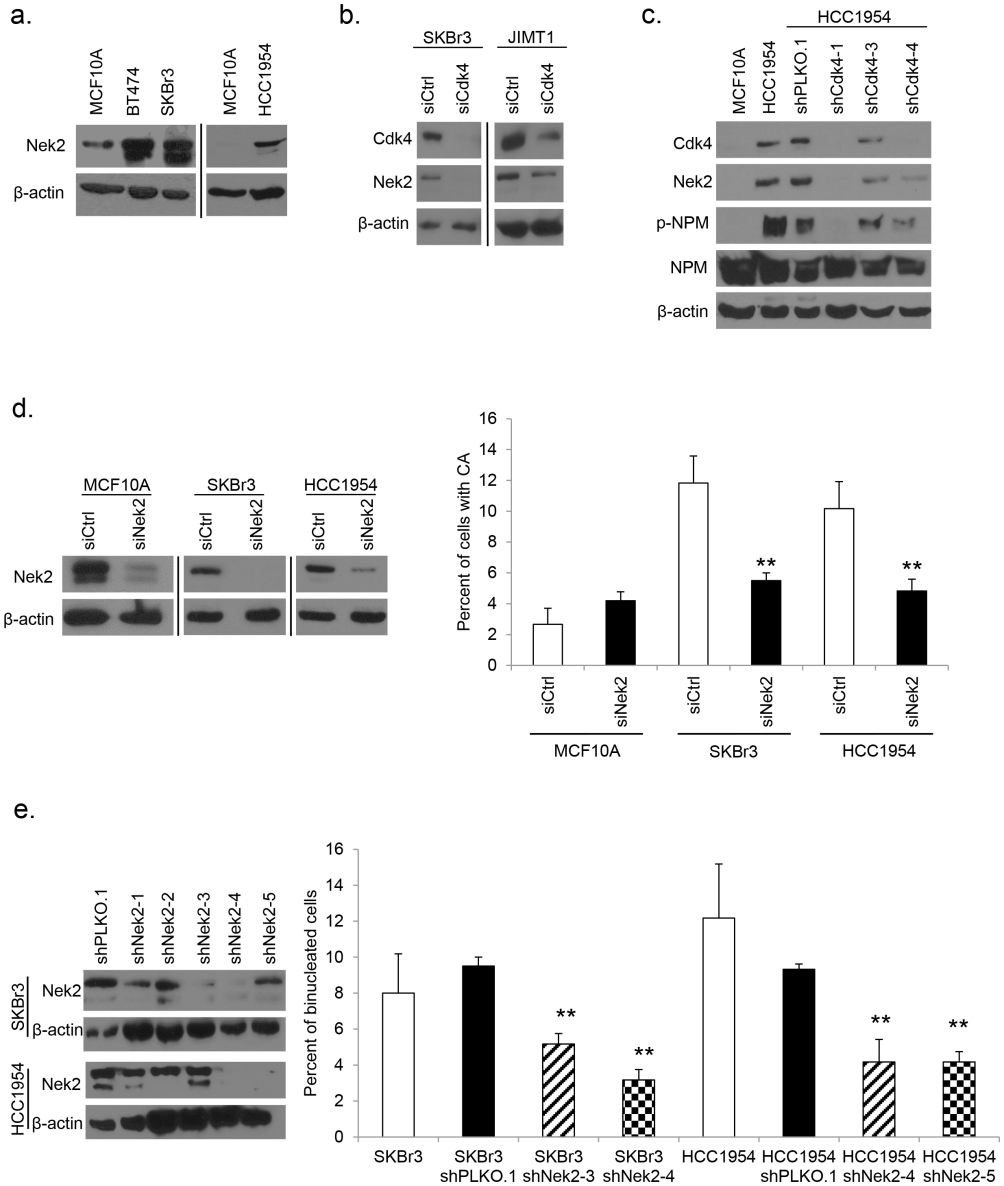
Binucleation and CA in Her2+ cells are mediated by Nek2. (a) Protein lysates from Figure 2a were used in western blots to detect levels of Nek2 in MCF10A and Her2+ breast cancer cell lines; β-actin was used as a loading control. Western blot results show two separate gels; different exposures are commensurate with protein abundance. (b) Western blotting was done in lysates collected from proliferating SKBr3 and JIMT1 cells transfected with siCdk4 constructs. Antibodies against Cdk4 and Nek2 were used; β-actin was used as a loading control. Western blot results show two separate gels; different exposures are commensurate with protein abundance. (c) Western blotting was done in lysates collected from serum arrested HCC1954 expressing shRNAs against Cdk4. Antibodies against Cdk4, Nek2, phospho-NPM, and NPM protein were used; β-actin was used as a loading control. (d) Transient transfection of siNek2 was performed in target cell lines; scrambled siRNA was used as a control. Knockdown was determined by western blotting using an antibody against Nek2; β-actin was used as a loading control. CA was measured as described in Figure 1a. Western blot results show two separate gels; different exposures are commensurate with protein abundance. (e) Lentiviral shPLKO.1 control and shNek2 vectors were used to infect SKBr3 and HCC1954 cells and create stable cell lines via puromycin selection. Independent lentiviral clones were screened in each cell line; knockdown was confirmed by western blot using an antibody against Nek2; β-actin was used as a loading control. The percentage of binucleation was compared in SKBr3 parental, shPLKO.1, and two independent shNek2 cell lines, and HCC1954 parental, shPLKO.1 control, and two independent shNek2 cell lines as described in Figure 3a. Statistical significance was addressed using a T-test (* = p≤0.05; ** = p≤0.01).

We followed up with Nek2 as an important target gene in our model, based on the abnormal binucleation phenotype, and found that Nek2 protein was overexpressed in the Her2+ cell lines investigated in comparison to MCF10A cells (Figure 4a). We transiently transfected siCdk4 constructs into two Her2+ cell lines to assay the level of Nek2 upon loss of Cdk4 under proliferating conditions; interestingly we found that knockdown of Cdk4 lead to reduction of Nek2 levels (Figure 4b). Upon probing control and shCdk4 expressing HCC1954 cells with antibodies against Nek2 we discovered that knockdown of Cdk4 resulted in a decrease of Nek2 protein expression in serum-arrested cells (Figure 4c). We also detected a decrease in the level of phosphorylated NPM in cells expressing shRNAs against Cdk4 (Figure 4c). This target provides an interesting avenue for further investigation.

These novel findings suggest a functional correlation between Cdk4 and the potential CA regulator, Nek2. Next, we performed qRT-PCR experiments under proliferating and serum starvation conditions to address the role of Cdk4 in regulating Nek2 at the transcriptional level. We found no significant difference in the amount of Nek2 mRNA in any of the cell lines investigated at either proliferation or quiescence, suggesting that the silencing of Cdk4 does not impact Nek2 at the transcriptional level (data not shown). To further pursue Nek2 as a mediator of CA, we transfected siRNA constructs into MCF10A, SKBr3, and HCC1954 cells and assessed the percentage of CA. The reduction of Nek2 by siRNA phenocopied loss of Cdk4 and reduced the percentage of CA found in Her2+ cells (Figure 4d).

Overexpression of recombinant active Nek2 in human cancer cells induces premature centriole splitting at G_1_/S, while still allowing cells to enter mitosis [53]. Deregulated Nek2 has also been associated with abnormalities in cytokinesis in mammary epithelial cells immortalized with SV40 large T antigen [56]. To elucidate a role for Nek2 in the observed binucleation phenotype of the Her2+ breast cancer model, we stained SKBr3 and HCC1954 shNek2 cells with antibodies against α-tubulin and DAPI in order to image the cytoskeleton and nucleus, respectively. This assay revealed that knocking down Nek2 reduced the percentage of binucleation in proliferating cells, as control shPLKO.1 cells maintained high levels of binucleation, while shNek2 cells showed significantly lower percentages (Figure 4e). This data shows that Nek2 mediates CA and binucleation in Her2+ breast cancer cells.

**Figure 5:**
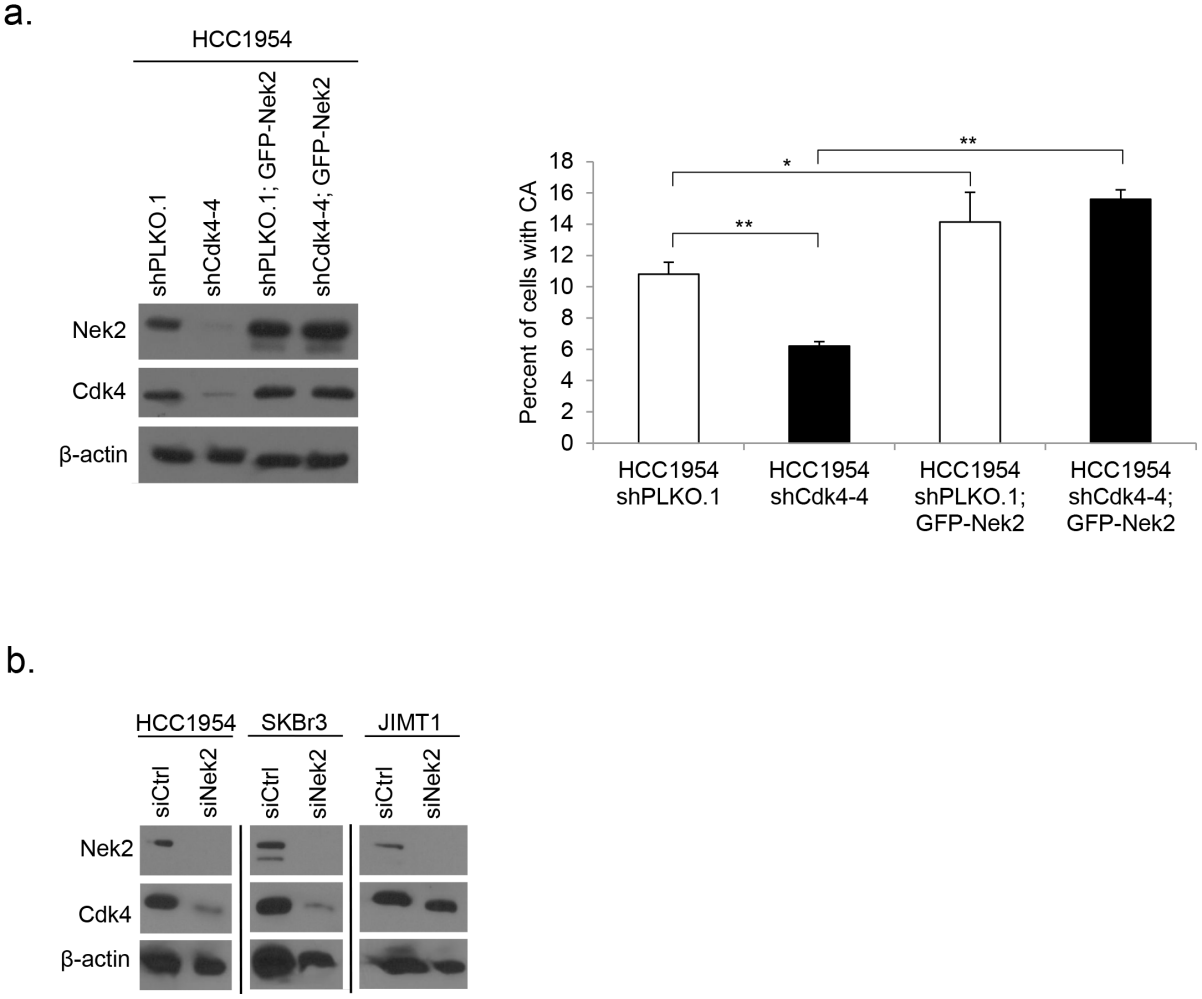
A potential signaling loop for Cdk4 and Nek2. (a) Cells were serum starved for 72 hours, and overexpression of GFP-Nek2 plasmid was confirmed by western blotting using antibodies against Nek2 and GFP; protein lysates were also probed with an antibody against Cdk4. The number of centrosomes was assayed as in Figure 1a. Statistical significance was addressed using a T-test (* = p≤0.05; ** = p≤0.01). (b) Western blotting was done on protein lysates collected from proliferating cells transfected with siNek2 constructs. Knockdown was confirmed using an antibody against Nek2; membranes were then probed with an antibody against; β-actin was used as a loading control. Western blot results show three separate gels; different exposures are commensurate with protein abundance.

This data suggests that Nek2 is possibly downstream of Cdk4 and important in inducing CA. To further address this possibility we attempted a rescue experiment by introducing an overexpression plasmid, GFP-Nek2, into HCC1954 cells expressing either shPLKO.1 or shCdk4. We were unable to obtain Nek2-overexpressing shCdk4-1 and shCdk4-3 cell populations, as these transfectants stopped proliferating. Nevertheless, we were able to establish stable populations of HCC1954 shPLKO.1 and shCdk4-4 cells and confirm overexpression of Nek2 via Western blot by probing for both Nek2 protein as well as GFP (Figure 5a). Interestingly, expression of Cdk4 protein was restored in cells overexpressing Nek2. The presence of GFP-Nek2 increased the percentage of CA in both control and HCC1954 shCdk4-4 cells compared to their relative controls (Figure 5a). To better understand a potential signaling pathway, we transfected siNek2 constructs into three Her2+ cell lines, HC1954, SKBr3, and JIMT1, and examined the levels of Cdk4 protein expression. We found HCC1954 and SKBr3 cells with confirmed Nek2 knockdown showed a marked reduction in Cdk4 expression. JIMT1 showed a slight reduction in Cdk4 upon knockdown of Nek2 (Figure 5b). We found no significant difference in the level of Cdk4 mRNA, suggesting that the silencing of Nek2 does not affect Cdk4 at the transcriptional level (data not shown). In this report we show that Nek2 plays a key role in identifying the mechanism behind CA and binucleation in a Her2+ breast cancer model.

**Figure 6:**
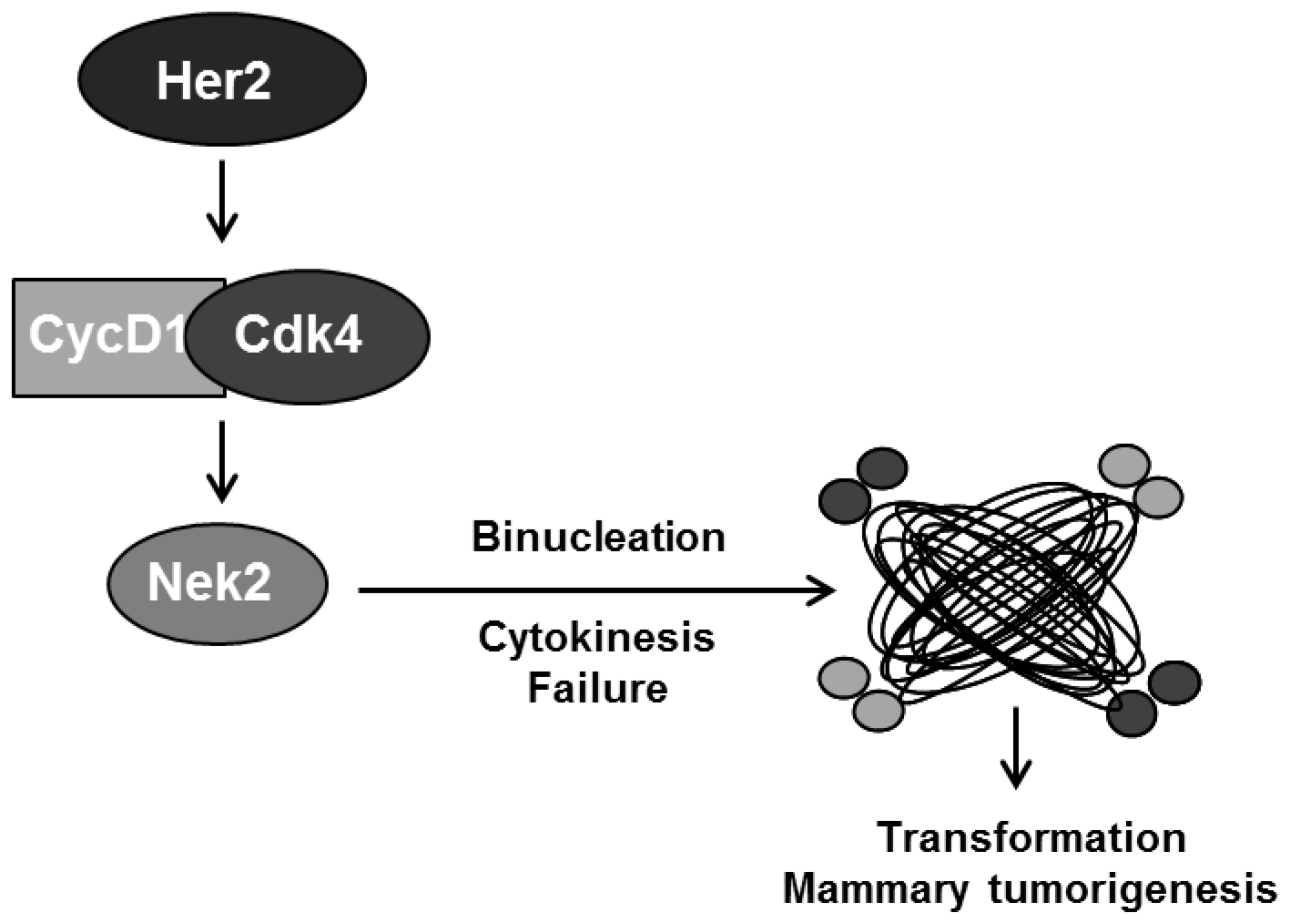
Working model. Our working model proposes that in a Her2+ breast cancer model overexpression of cyclin D1/Cdk4 leads to an abundance of Nek2. Based on our results and the results of others, overexpression of Nek2 could drive binucleation through failed cytokinesis, leading to CA and potentially transformation and mammary tumorigenesis.

## Discussion

A major proportion of human tumor cells harbor centrosome abnormalities. These aberrant phenotypes have been hypothesized to generate multipolar mitoses, microtubule nucleation errors, aneuploidy, chromosome instability, and even tumorigenesis. Understanding whether CA plays a role in breast tumorigenesis requires identification of the pathways and molecules that directly signal CA. Once such pathways and molecules are identified, their inhibition and/or overexpression will lead to a better understanding of their role in transformation. Our early work demonstrated that the Ras oncogene signals CA through the MAPK pathway, while other Ras-dependent pathways do not act on CA [58], [59]. We also showed that Ras is able to trigger CA in premalignant mammary epithelial lesions, whereas c-Myc is unable to do so [28]. These experiments indicate that CA is specific to certain oncogenic and signaling pathways and that CA may drive early mammary tumorigenesis.

Adding to our body of work detailing oncogene and tumor suppressor specific contributions to CA [26], [28], we studied CA in a Her2-positive breast cancer model. Previous studies addressing the involvement of cell cycle regulators in the centrosome cycle demonstrate that the loss of E2F3 and p53 deregulated Cdk2 activity, resulting in CA [50], [60]. Recent work by our lab showed Cdk2 and Cdk4 are key regulators of CA in p53-null cells [26], and that silencing of cyclin D1/Cdk4 inhibits CA driven by H-Ras^G12V^, or H-Ras^G12V^ and c-Myc [28]. The results presented in this report clearly show that Cdk4 is more influential than Cdk2 in mediating CA in Her2+ breast cancer cells. Importantly, Cdk4 inhibition abrogates CA without significantly interfering with the cell cycle, suggesting that a unique function of Cdk4 is to signal CA in a subset of Her2+ breast cancer cells. This observation is not specific to Her2 overexpression, as inhibition of Cdk4 suppressed CA in p53-null cells and in MCF10A expressing Ras, or Ras and Myc, without significantly affecting the cell cycle [26], [51]. The implications of this result are potentially important to the treatment of breast cancer patients. We speculate that inhibiting Cdk4 in Her2+ breast tumors can suppress some malignant characteristics of tumor cells such as CA and the active generation of aneuploidy.

Our past work showed that Cdk4 is essential for centriole reduplication, an important intermediate to CA [26]. The novelty of the studies described here revolves around Cdk4 signaling cytokinesis defects, binucleation, and CA. While the Cdks are typically related to CA through the deregulation of the centrosome cycle [26], [50], [61], so far, no one has shown that Cdk4 can influence aspects of cytokinesis. Interestingly, we found that knockdown of Cdk4 leads to a reduction in the level of Nek2 protein expression, which leads to a reduction in the percentage of binucleation and CA in Her2+ cells. This observation suggests a novel molecular mechanism where Nek2 can mediate some of the oncogenic functions of Cdk4. While cleavage failure is not sufficient to establish CA in untransformed cells, it has been shown that in transformed CHO p53^−/−^ cells several rounds of cleavage failure caused a small increase in CA that does not persist at later passages [18].

Compelling data indicates high levels of the centrosomal kinase Nek2 protein in cell lines derived from breast, cervical, and prostate carcinomas. Overexpression of Nek2 in immortalized breast cancer cells induces aneuploidy through multinucleated cells, and these cells are typically associated with CA [56]. Additionally, transient overexpression of kinase-active Nek2 induces premature centrosome splitting [53]. Nek2 protein can be regulated both temporally and spatially in various ways, including through transcription, post-translational modifications, and protein-protein interactions. The abundance of Nek2 is also managed by cell cycle-dependent protein degradation; it is normally targeted for proteasomal degradation following ubiquitylation facilitated by the anaphase promoting complex/cyclosome (APC/C). Failure to appropriately degrade Nek2 could increase stability and abundance within the cell [62]. It is plausible, based on the role of Nek2 in centrosome separation and microtubule organization, that overexpression of Nek2 could lead to CA via binucleation, potentially through a defect in cytokinesis. Perhaps the most direct evidence for Nek2’s role in cytokinesis comes from the *Drosophila* model system. At wild-type levels DmNek2 localizes to the midbody, and overexpression of DmNek2 causes a decrease in normal cytokinesis and an increase in tetraploid cells [63]. There is sufficient evidence of centrosomal aberrations leading to mitotic defects, and there is a growing body of work suggesting that Nek2 is one of the molecules that maintains mitotic events. In light of these findings, we propose a model where the overabundance of Nek2 in Her2+ cells is caused by deregulated cyclin D1/Cdk4 activity and that in turn, Nek2 is required to facilitate some of the abnormal mitotic functions triggered by cyclin D1/Cdk4 (Figure 5). Experiments attempting to rescue CA in cells stably silenced for Cdk4 were inconclusive. The only shCdk4 cells overexpressing Nek2 that proliferated were those that re-expressed Cdk4. This result could be interpreted as evidence of an interesting signaling loop, wherein high levels of Nek2 can positively regulate expression of Cdk4. This is suggested by the reduction in Cdk4 levels upon knockdown of Nek2. Alternatively, Nek2 overexpression in cells lacking Cdk4 might impose cell cycle blocks and impair cell proliferation.

Discovering that inhibition of Nek2 or Cdk4 diminishes CA in breast cancer cells, and showing that silencing of Cdk4 leads to reduced Nek2 overexpression is important, as both molecules have been shown to mediate mammary epithelial transformation [41], [64]. As demonstrated in this manuscript, the inhibition of Cdk4 or Nek2 prevents CA in Her2+ cells, which is indicative of the important role of CA in mammary transformation. This manuscript furthers the understanding of the role that CA plays in breast cancers by identifying Cdk4 and Nek2 as mediators of CA in Her2+ breast cancer cells, and by identifying binucleation as a major mechanism generating CA in breast cancers. This has potential translational relevance because CA may be a driver of breast cancer biogenesis, exemplified by the presence of CA in pre-malignant mammary epithelial lesions in humans and in mice expressing various oncogenes. On the other hand, aneuploidy generated by CA can also drive resistance to chemotherapeutic agents. Thus, further studies are needed to establish whether inhibition of CA via the Cdk4-Nek2 pathway will improve the clinical outcome of breast cancer patients.

## Supporting Information

Table S1 **siRNA sequences targeting the indicated genes.**
(DOCX)

## References

[1] Boveri T (1914) Zur Frage der Entstehung Maligner Tumoren (Jena: Fischer Verlag, 1914 (English translation by M. Boveri reprinted as The Origin of malignant tumors, The Williams and Wilkins Co., Baltimore), 1929.

[2] Sluder G, Hinchcliffe EH (1999) Control of centrosome reproduction: the right number at the right time. Biol Cell 91: 413–427. 10519003

[3] Doxsey S (2002) Duplicating dangerously: linking centrosome duplication and aneuploidy. Mol Cell 10: 439–440. 12408813 10.1016/s1097-2765(02)00654-8

[4] Pihan GA, Purohit A, Wallace J, Knecht H, Woda B, et al (1998) Centrosome defects and genetic instability in malignant tumors. Cancer Research 58: 3974–3985. 9731511

[5] Lingle WL, Barrett SL, Negron VC, D'Assoro AB, Boeneman K, et al (2002) Centrosome amplification drives chromosomal instability in breast tumor development. Proceedings of the National Academy of Sciences of the United States of America 99: 1978–1983. 11830638 10.1073/pnas.032479999 PMC122305

[6] Carroll PE, Okuda M, Horn HF, Biddinger P, Stambrook PJ, et al (1999) Centrosome hyperamplification in human cancer: chromosome instability induced by p53 mutation and/or Mdm2 overexpression. Oncogene 18: 1935–1944. 10208415 10.1038/sj.onc.1202515

[7] Zyss D, Gergely F (2009) Centrosome function in cancer: guilty or innocent? Trends Cell Biol 19: 334–346. 19570677 10.1016/j.tcb.2009.04.001

[8] Lingle WL, Lutz WH, Ingle JN, Maihle NJ, Salisbury JL (1998) Centrosome hypertrophy in human breast tumors: implications for genomic stability and cell polarity. Proc Natl Acad Sci U S A 95: 2950–2955. 9501196 10.1073/pnas.95.6.2950 PMC19675

[9] Guo HQ, Gao M, Ma J, Xiao T, Zhao LL, et al (2007) Analysis of the cellular centrosome in fine-needle aspirations of the breast. Breast Cancer Res 9: R48. 17662154 10.1186/bcr1752 PMC2206724

[10] Pihan GA, Wallace J, Zhou Y, Doxsey SJ (2003) Centrosome abnormalities and chromosome instability occur together in pre-invasive carcinomas. Cancer Res 63: 1398–1404. 12649205

[11] Lingle WL, Barrett SL, Negron VC, D'Assoro AB, Boeneman K, et al (2002) Centrosome amplification drives chromosomal instability in breast tumor development. Proc Natl Acad Sci U S A 99: 1978–1983. 11830638 10.1073/pnas.032479999 PMC122305

[12] Basto R, Brunk K, Vinadogrova T, Peel N, Franz A, et al (2008) Centrosome amplification can initiate tumorigenesis in flies. Cell 133: 1032–1042. 18555779 10.1016/j.cell.2008.05.039 PMC2653712

[13] Castellanos E, Dominguez P, Gonzalez C (2008) Centrosome dysfunction in Drosophila neural stem cells causes tumors that are not due to genome instability. Curr Biol 18: 1209–1214. 18656356 10.1016/j.cub.2008.07.029

[14] Weaver BA, Cleveland DW (2007) Aneuploidy: instigator and inhibitor of tumorigenesis. Cancer Res 67: 10103–10105. 17974949 10.1158/0008-5472.CAN-07-2266 PMC3132555

[15] Schliekelman M, Cowley DO, O'Quinn R, Oliver TG, Lu L, et al (2009) Impaired Bub1 function in vivo compromises tension-dependent checkpoint function leading to aneuploidy and tumorigenesis. Cancer Res 69: 45–54. 19117986 10.1158/0008-5472.CAN-07-6330 PMC4770788

[16] Ganem NJ, Godinho SA, Pellman D (2009) A mechanism linking extra centrosomes to chromosomal instability. Nature 460: 278–282. 19506557 10.1038/nature08136 PMC2743290

[17] Fukasawa K (2007) Oncogenes and tumour suppressors take on centrosomes. Nat Rev Cancer 7: 911–924. 18004399 10.1038/nrc2249

[18] Krzywicka-Racka A, Sluder G (2011) Repeated cleavage failure does not establish centrosome amplification in untransformed human cells. J Cell Biol 194: 199–207. 21788368 10.1083/jcb.201101073 PMC3144409

[19] Fujiwara T, Bandi M, Nitta M, Ivanova EV, Bronson RT, et al (2005) Cytokinesis failure generating tetraploids promotes tumorigenesis in p53-null cells. Nature 437: 1043–1047. 16222300 10.1038/nature04217

[20] Fukasawa K (2005) Centrosome amplification, chromosome instability and cancer development. Cancer Lett 230: 6–19. 16253756 10.1016/j.canlet.2004.12.028

[21] Gopinathan L, Ratnacaram CK, Kaldis P (2011) Established and novel Cdk/cyclin complexes regulating the cell cycle and development. Results Probl Cell Differ 53: 365–389. 21630153 10.1007/978-3-642-19065-0_16

[22] Satyanarayana A, Kaldis P (2009) Mammalian cell-cycle regulation: several Cdks, numerous cyclins and diverse compensatory mechanisms. Oncogene 28: 2925–2939. 19561645 10.1038/onc.2009.170

[23] Hartwell LH, Weinert TA (1989) Checkpoints: controls that ensure the order of cell cycle events. Science 246: 629–634. 2683079 10.1126/science.2683079

[24] D'Assoro AB, Lingle WL, Salisbury JL (2002) Centrosome amplification and the development of cancer. Oncogene 21: 6146–6153. 12214243 10.1038/sj.onc.1205772

[25] Harrison MK, Adon AM, Saavedra HI (2011) The G1 phase Cdks regulate the centrosome cycle and mediate oncogene-dependent centrosome amplification. Cell Div 6: 2. 21272329 10.1186/1747-1028-6-2 PMC3038874

[26] Adon AM, Zeng X, Harrison MK, Sannem S, Kiyokawa H, et al (2010) Cdk2 and Cdk4 regulate the centrosome cycle and are critical mediators of centrosome amplification in p53-null cells. Mol Cell Biol 30: 694–710. 19933848 10.1128/MCB.00253-09 PMC2812235

[27] Nelsen CJ, Kuriyama R, Hirsch B, Negron VC, Lingle WL, et al (2005) Short term cyclin D1 overexpression induces centrosome amplification, mitotic spindle abnormalities, and aneuploidy. J Biol Chem 280: 768–776. 15509582 10.1074/jbc.M407105200

[28] Zeng X, Shaikh FY, Harrison MK, Adon AM, Trimboli AJ, et al (2010) The Ras oncogene signals centrosome amplification in mammary epithelial cells through cyclin D1/Cdk4 and Nek2. Oncogene 29: 5103–5112. 20581865 10.1038/onc.2010.253 PMC2972189

[29] Harari D, Yarden Y (2000) Molecular mechanisms underlying ErbB2/HER2 action in breast cancer. Oncogene 19: 6102–6114. 11156523 10.1038/sj.onc.1203973

[30] Yarden Y, Sliwkowski MX (2001) Untangling the ErbB signalling network. Nat Rev Mol Cell Biol 2: 127–137. 11252954 10.1038/35052073

[31] Yu Q, Sicinska E, Geng Y, Ahnstrom M, Zagozdzon A, et al (2006) Requirement for CDK4 kinase function in breast cancer. Cancer Cell 9: 23–32. 16413469 10.1016/j.ccr.2005.12.012

[32] Okuda M, Horn HF, Tarapore P, Tokuyama Y, Smulian AG, et al (2000) Nucleophosmin/B23 is a target of CDK2/cyclin E in centrosome duplication. Cell 103: 127–140. 11051553 10.1016/s0092-8674(00)00093-3

[33] Lacey KR, Jackson PK, Stearns T (1999) Cyclin-dependent kinase control of centrosome duplication. Proceedings of the National Academy of Sciences of the United States of America 96: 2817–2822. 10077594 10.1073/pnas.96.6.2817 PMC15852

[34] Matsumoto Y, Hayashi K, Nishida E (1999) Cyclin-dependent kinase 2 (Cdk2) is required for centrosome duplication in mammalian cells. Current Biology 9: 429–432. 10226033 10.1016/s0960-9822(99)80191-2

[35] Hinchcliffe EH, Li C, Thompson EA, Maller JL, Sluder G (1999) Requirement of Cdk2-cyclin E activity for repeated centrosome reproduction in Xenopus egg extracts. Science 283: 851–854. 9933170 10.1126/science.283.5403.851

[36] D'Assoro AB, Busby R, Suino K, Delva E, Almodovar-Mercado GJ, et al (2004) Genotoxic stress leads to centrosome amplification in breast cancer cell lines that have an inactive G1/S cell cycle checkpoint. Oncogene 23: 4068–4075. 15064746 10.1038/sj.onc.1207568

[37] Hanashiro K, Kanai M, Geng Y, Sicinski P, Fukasawa K (2008) Roles of cyclins A and E in induction of centrosome amplification in p53-compromised cells. Oncogene. 10.1038/onc.2008.161 PMC2574884 18490919

[38] Tarapore P, Horn HF, Tokuyama Y, Fukasawa K (2001) Direct regulation of the centrosome duplication cycle by the p53-p21Waf1/Cip1 pathway. Oncogene 20: 3173–3184. 11423967 10.1038/sj.onc.1204424

[39] Duensing A, Liu Y, Tseng M, Malumbres M, Barbacid M, et al (2006) Cyclin-dependent kinase 2 is dispensable for normal centrosome duplication but required for oncogene-induced centrosome overduplication. Oncogene 25: 2943–2949. 16331279 10.1038/sj.onc.1209310 PMC2225596

[40] Ray D, Terao Y, Christov K, Kaldis P, Kiyokawa H (2011) Cdk2-null mice are resistant to ErbB-2-induced mammary tumorigenesis. Neoplasia 13: 439–444. 21532884 10.1593/neo.101704 PMC3084620

[41] Reddy HK, Mettus RV, Rane SG, Grana X, Litvin J, et al (2005) Cyclin-dependent kinase 4 expression is essential for neu-induced breast tumorigenesis. Cancer Res 65: 10174–10178. 16288002 10.1158/0008-5472.CAN-05-2639

[42] Lee RJ, Albanese C, Fu M, D'Amico M, Lin B, et al (2000) Cyclin D1 is required for transformation by activated Neu and is induced through an E2F-dependent signaling pathway. Mol Cell Biol 20: 672–683. 10611246 10.1128/mcb.20.2.672-683.2000 PMC85165

[43] Yu Q, Geng Y, Sicinski P (2001) Specific protection against breast cancers by cyclin D1 ablation. Nature 411: 1017–1021. 11429595 10.1038/35082500

[44] Roberts PJ, Bisi JE, Strum JC, Combest AJ, Darr DB, et al (2012) Multiple roles of cyclin-dependent kinase 4/6 inhibitors in cancer therapy. J Natl Cancer Inst 104: 476–487. 22302033 10.1093/jnci/djs002 PMC3309128

[45] Schneeweiss A, Sinn HP, Ehemann V, Khbeis T, Neben K, et al (2003) Centrosomal aberrations in primary invasive breast cancer are associated with nodal status and hormone receptor expression. Int J Cancer 107: 346–352. 14506732 10.1002/ijc.11408

[46] Montagna C, Andrechek ER, Padilla-Nash H, Muller WJ, Ried T (2002) Centrosome abnormalities, recurring deletions of chromosome 4, and genomic amplification of HER2/neu define mouse mammary gland adenocarcinomas induced by mutant HER2/neu. Oncogene 21: 890–898. 11840334 10.1038/sj.onc.1205146

[47] Salisbury JL, D'Assoro AB, Lingle WL (2004) Centrosome amplification and the origin of chromosomal instability in breast cancer. J Mammary Gland Biol Neoplasia 9: 275–283. 15557800 10.1023/B:JOMG.0000048774.27697.30

[48] Gao Y, Niu Y, Wang X, Wei L, Zhang R, et al (2011) Chromosome aberrations associated with centrosome defects: a study of comparative genomic hybridization in breast cancer. Hum Pathol 42: 1693–1701. 21531002 10.1016/j.humpath.2010.12.027

[49] Bouchet BP, Bertholon J, Falette N, Audoynaud C, Lamblot C, et al (2007) Paclitaxel resistance in untransformed human mammary epithelial cells is associated with an aneuploidy-prone phenotype. Br J Cancer 97: 1218–1224. 17968427 10.1038/sj.bjc.6603936 PMC2360475

[50] Saavedra HI, Maiti B, Timmers C, Altura R, Tokuyama Y, et al (2003) Inactivation of E2F3 results in centrosome amplification. Cancer Cell 3: 333–346. 12726860 10.1016/s1535-6108(03)00083-7 PMC3033013

[51] Zeng X, Shaikh FY, Harrison MK, Adon AM, Trimboli AJ, et al.. (2010) The Ras oncogene signals centrosome amplification in mammary epithelial cells through cyclin D1/Cdk4 and Nek2. Oncogene. 10.1038/onc.2010.253 PMC2972189 20581865

[52] Anderhub SJ, Kramer A, Maier B (2012) Centrosome amplification in tumorigenesis. Cancer Lett. 10.1016/j.canlet.2012.02.006 22342684

[53] Fry AM, Meraldi P, Nigg EA (1998) A centrosomal function for the human Nek2 protein kinase, a member of the NIMA family of cell cycle regulators. Embo J 17: 470–481. 9430639 10.1093/emboj/17.2.470 PMC1170398

[54] Chen D, Pacal M, Wenzel P, Knoepfler PS, Leone G, et al (2009) Division and apoptosis of E2f-deficient retinal progenitors. Nature 462: 925–929. 20016601 10.1038/nature08544 PMC2813224

[55] Chong JL, Wenzel PL, Saenz-Robles MT, Nair V, Ferrey A, et al (2009) E2f1–3 switch from activators in progenitor cells to repressors in differentiating cells. Nature 462: 930–934. 20016602 10.1038/nature08677 PMC2806193

[56] Hayward DG, Clarke RB, Faragher AJ, Pillai MR, Hagan IM, et al (2004) The centrosomal kinase Nek2 displays elevated levels of protein expression in human breast cancer. Cancer Res 64: 7370–7376. 15492258 10.1158/0008-5472.CAN-04-0960

[57] Tokuyama Y, Horn HF, Kawamura K, Tarapore P, Fukasawa K (2001) Specific phosphorylation of nucleophosmin on Thr(199) by cyclin-dependent kinase 2-cyclin E and its role in centrosome duplication. Journal of Biological Chemistry 276: 21529–21537. 11278991 10.1074/jbc.M100014200

[58] Saavedra HI, Fukasawa K, Conn CW, Stambrook PJ (1999) MAPK mediates RAS-induced chromosome instability. J Biol Chem 274: 38083–38090. 10608877 10.1074/jbc.274.53.38083

[59] Saavedra HI, Knauf JA, Shirokawa JM, Wang J, Ouyang B, et al (2000) The RAS oncogene induces genomic instability in thyroid PCCL3 cells via the MAPK pathway. Oncogene 19: 3948–3954. 10951588 10.1038/sj.onc.1203723

[60] Fukasawa K, Choi T, Kuriyama R, Rulong S, Vande Woude GF (1996) Abnormal centrosome amplification in the absence of p53. Science 271: 1744–1747. 8596939 10.1126/science.271.5256.1744

[61] Fukasawa K (2008) p53, cyclin-dependent kinase and abnormal amplification of centrosomes. Biochim Biophys Acta. 10.1016/j.bbcan.2008.04.002 PMC2647860 18472015

[62] Hayward DG, Fry AM (2006) Nek2 kinase in chromosome instability and cancer. Cancer Lett 237: 155–166. 16084011 10.1016/j.canlet.2005.06.017

[63] Prigent C, Glover DM, Giet R (2005) Drosophila Nek2 protein kinase knockdown leads to centrosome maturation defects while overexpression causes centrosome fragmentation and cytokinesis failure. Exp Cell Res 303: 1–13. 15572022 10.1016/j.yexcr.2004.04.052

[64] Tsunoda N, Kokuryo T, Oda K, Senga T, Yokoyama Y, et al (2009) Nek2 as a novel molecular target for the treatment of breast carcinoma. Cancer Sci 100: 111–116. 19038001 10.1111/j.1349-7006.2008.01007.x PMC11158353

