# Supplementary material for: Cdk4 and Nek2 Signal Binucleation and Centrosome Amplification in a Her2+ Breast Cancer Model
Source: PLoS One. 2013 Jun 11;8(6):e65971. doi: 10.1371/journal.pone.0065971 (PMC3679029; doi:10.1371/journal.pone.0065971)
Supplement: Table S1 — siRNA sequences targeting the indicated genes. (DOCX) [file pone.0065971.s001.docx]

| siRNA | 5’-3’Sequence | 3’-5’Sequence |
| --- | --- | --- |
| siCdk4-3 | AAGUAAUCUCUGUAGAAAGAUGGAGGA | TTCAUUAGAGACAUCUUUCUACCUC |
| siCdk4-4 | ACCUUCAUCCUUAUGUAGAUAAGAGUG | TGGAAGUAGGAAUACAUCUAUUCUC |
| siCdk2-4 | UGCCUAAACCCUAACUUCUAACUCCUA | ACGGAUUUGGGAUUGAAGAUUGAGG |
| siCdk2-7 | CCCAGAUUCCUCAUGCCUGUAGAAGGG | GGGUCUAAGGAGUACGGACAUCUUC |
| siNek2 | UUCAACAGAAGGUCGAUGGUAAUCCUU | AAGUUGUCUUCCAGCUACCAUUAGG |

Table S1. siRNA sequences targeting the indicated genes.

**TABLE S1**
